# A case series of children with adenovirus pneumonia: three-year experiences in a tertiary PICU

**DOI:** 10.1186/s12887-020-02269-5

**Published:** 2020-08-10

**Authors:** Jingyi Shi, Yiping Zhou, Fei Wang, Chunxia Wang, Huijie Miao, Ting Sun, Yijun Shan, Yun Cui, Yucai Zhang

**Affiliations:** Department of Critical Care Medicine, Shanghai Children’s Hospital; Institute of Pediatric Critical Care, Shanghai Jiao Tong University, No.355 Luding Road, Putuo District, Shanghai, 200062 China

**Keywords:** Adenovirus pneumonia, Outcome, Mortality, Pediatric intensive care unit (PICU)

## Abstract

**Background:**

Describe the outcome of adenovirus pneumonia in a pediatric intensive care unit (PICU) over a 3-year period, to identify the risk factors that may be associated with worse outcome.

**Methods:**

A retrospective observational study was performed in the PICU of children’s hospital in Shanghai from July 2016 to June 2019. Sixty-seven children over 29 days to 14 years old with adenovirus pneumonia who were admitted to PICU with acute hypoxemic respiratory failure were included in this study. The primary outcome was hospital mortality, and secondary outcomes were hospital and PICU length of stay (LOS), and risk factors of worse outcome.

**Results:**

Of 67 children with severe adenovirus pneumonia, the hospital mortality was 16.42% (11/67) and 28-day mortality was 14.93% (10/67). Median Pediatric Risk of Mortality III (PRISM III) score at admission was 13 (interquartile range [IQR], 10–15). Median PICU LOS stay was 11 days (8-18d) and hospital LOS was 22 days (16-31d). Among children with extracorporeal membrane oxygenation (*n* = 9), 6 cases survived and 3 cases died. The patients who need renal replacement therapy, neuromuscular blockade, parenteral nutrition, and packed red blood cell perfusion had higher hospital mortality (*p* < 0.001, *p* = 0.041, *p* = < 0.001, *p* = 0.012, respectively). Multivariate logistic analysis indicated that liver dysfunction and nosocomial infection were associated with high risk of mortality.

**Conclusions:**

The hospital mortality of adenovirus pneumonia in our PICU was 16.42%. Patients complicated liver dysfunction and co-infection & nosocomial infection were associated with poor outcome.

## Background

Adenovirus is a common pathogen of respiratory tract infection in all age groups. The clinical course of this virus infection in immunocompetent patients is usually self-limited. However, adenovirus infection can cause significant morbidity and mortality in young children or immunocompromised persons [1, 2]. Moreover, adenovirus has been increasingly found to be involved in sporadic cases and outbreaks of community acquired pneumonia (CAP) in infants and young children [3–5]. In some patients adenovirus infection cause severe pneumonia, myocarditis, hepatitis, encephalitis, and disseminated disease [2], which may quickly lead to refractory respiratory failure, acute respiratory distress syndrome (ARDS), and multiple organ dysfunction syndrome (MODS). If patients did not receive timely treatment, the mortality rate is over 50% had been described [3, 6]. Unfortunately, no effective antivirals or vaccines available for the prevention or treatment of adenovirus in children and adults either. Although Cidofovir reported to reduce the adenovirus load and to improve some series survivals, has not widely used in children yet. So, severe adenovirus pneumonia continued to provide pediatric intensive care unit (PICU) challenges.

The management of refractory hypoxic respiratory failure / ARDS seems to be improving in severe infection [7, 8]. Recently, limited studies reported that blood hemofiltration and ECMO were potential effective support for severe adenovirus pneumonia. However, the outcome is still far from satisfactory [9–12]. Furthermore, there is little information available for identifying risk factors for morbidity and mortality with severe adenovirus pneumonia in PICU [13].

Based on Lee and colleague’s study, adenovirus accounts for 5 to 10% of pediatric respiratory tract infection [14]. More recently, the incidence of pediatric adenoviral pneumonia has increased in some parts of China mainland [15]. The National Health Commission of China has issued the diagnosis and treatment of adenoviral pneumonia in children (2019) (http://www.nhc.gov.cn/yzygj/s7653p/201906/ab8ec27548ea48f793734e8d09c8d42c.shtml) recommended that children with severe illness should apply broad-spectrum antibiotics, glucocorticoids, bronchoscopy and mechanical ventilation. The indications of extracorporeal membrane lung (ECMO) and blood purification need to be carefully evaluated. Therefore, this retrospective observational study was conducted to better describe the clusters therapy strategies and outcomes of adenovirus infection in PICU.

## Methods

### Study design and inclusion criteria

We performed a retrospective analysis of prospectively collected data of patients with severe adenovirus pneumonia admitted to a 36-bed PICU in a tertiary university hospital (Shanghai Children’s Hospital, Shanghai Jiao Tong University, China) between July 2016 and June 2019. All patients with pneumonia were initially screened with rapid respiratory virus assay including respiratory syncytial virus, adenovirus, influenza virus and coxsackie virus with nasopharyngeal swab at PICU admission. If rapid assay screen was negative, the deeper respiratory secretions obtained via endotracheal tube or bronchoalveolar lavage collected by bronchoscopy were tested by real-time polymerase chain reaction (RT-PCR). The inclusion criteria were an age of 29 days to 14 years old. Adenovirus pneumonia was confirmed by a positive RT-PCR from respiratory secretions as well as chest X-ray. The exclusion included:1) Patient was hospital acquired adenovirus pneumonia;2) Children had been admitted to other hospital within the last 3 days prior to the present admission; and 3) Children re-admitted to the PICU without 7 days symptom-free period. The study was approved by the ethics committee of Hospital (Approval number: 2016R007-E01). Informed consent was waived because of its retrospective design.

### Observational variables

The clinical course of each patient was obtained through computerized medical record database at hospital. Patient outcomes were grouped into two categories: survivors and non-survivors. The primary end point was hospital mortality. Key secondary outcomes included 28-day mortality, length of PICU stay and hospital stay, duration of mechanical ventilation and ventilator parameters, the clusters of therapy strategies: extracorporeal membrane oxygenation [ECMO] applied for refractory shock or refractory hypoxic respiratory failure, continuous renal replacement therapy or renal replacement therapy [CRRT/RRT] applied for fluid overload or acute kidney injury, prone position ventilation applied when the ratio of PaO_2_/FiO_2_ lower than 150 mmHg, and neuromuscular blockade applied when the ratio of PaO_2_/FiO_2_ lower than 150 mmHg as well as the peak inspiration pressure higher than 27cmH_2_O. And also, the vasoactive and steroids use, IV immunoglobulin, packed red blood cell perfusion, parenteral nutrition and etc. were recorded respectively.

The parameters including age, gender, pediatric risk of mortality III (PRISM III), the ratio of PaO_2_/FiO_2_, lung dynamic compliance (Cdyn), cardiac index (CI), mean arterial pressure (MAP), co-morbidities, secondary infection pathogen were collected. We also collect blood gas values and transcutaneous saturations. The biochemical parameters for organ functions (total bilirubin [TBIL]; lactic acid [LA]; serum creatinine [sCr]; etc.), Above laboratory indexes were collected from the first test within 24 h PICU admission. The laboratory indexes include white blood cell, platelet counts (PLT), natural kill cell (NK), cytokines and T lymphocytes series at within 24 h and after 7 days PICU admission.

### Statistical analysis

Patient’s characteristics and outcomes were summarized as median (interquartile range, IQR) for variables and percentage for categorical variables. Mann-Whitney *U* test was used to compare the continuous variables with abnormally distributed data The *Fisher’s* exact test or *chi*-square test was used to compare the categorical data. Adjusted odd ratios (ORs) were estimated by multivariate logistic regression models including the variables with significant difference obtained from group comparison. A value of *P* < 0.05 was considered statistically significant. Data analyses were performed using Statistical analyses were performed using STATA 15.0 MP (College Station, Texas, USA).

## Results

### Baseline characteristics

**Fig. 1:**
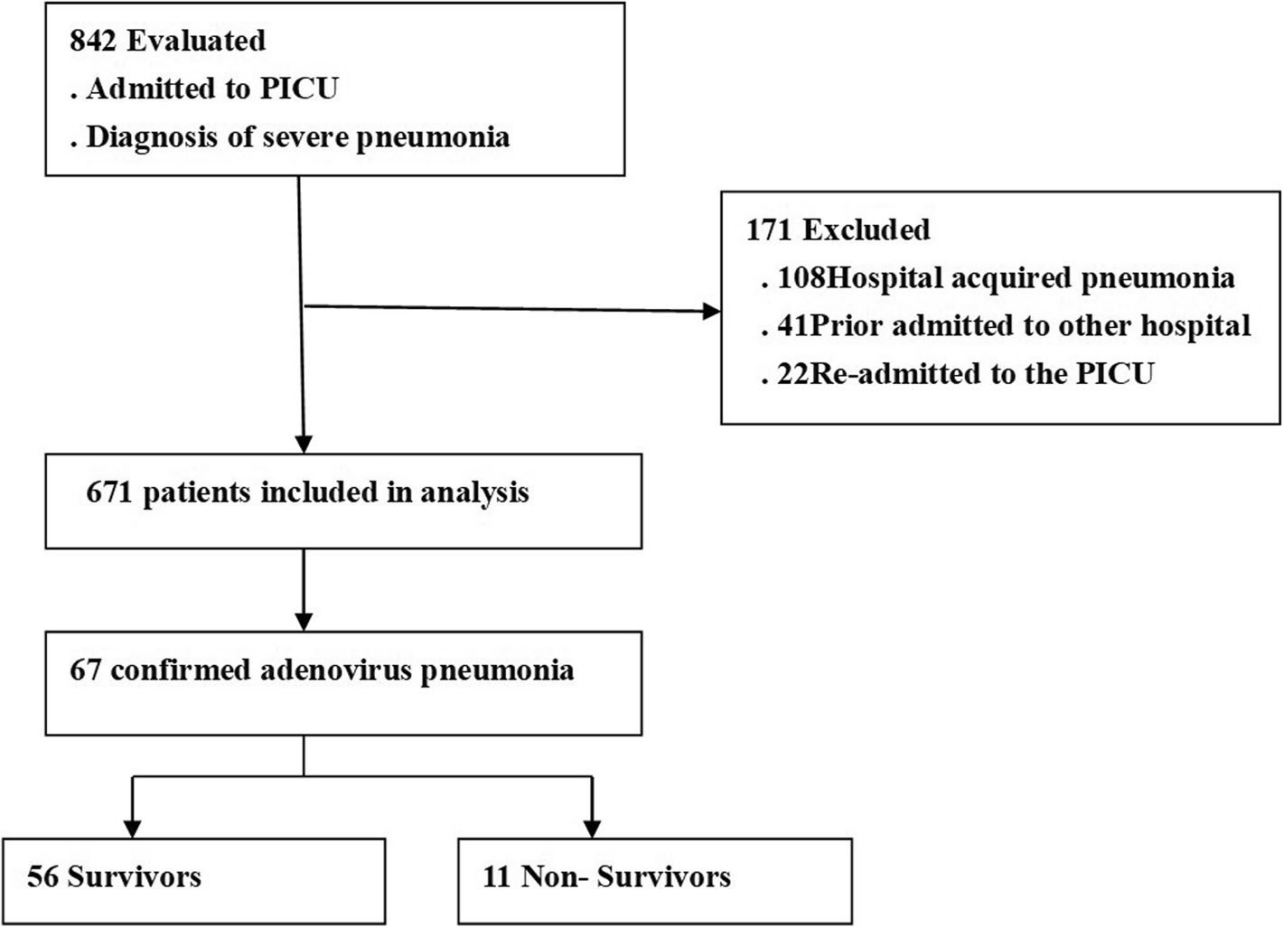
Patient enrolment and Study profile

Of 842 patients with pneumonia that requires PICU admission during the study period, 671 cases were community-acquired pneumonia (CAP). Among CAP, 67 with primary adenovirus infection were identified, and adenovirus accounted for 9.99% for all severe CAP admission. The patient enrolment and study profile were shown in Fig. 1. Among included patients, the median age was 18 (10, 38.5) months and 40 patients (59.7%) were male. Children aged < 24 months accounted for 83.6% (56/67) of all cases. The main characteristics at initial PICU admission between survivors and non-survivors were summarized in Table 1.

**Table 1:** Baseline characteristics at PICU admission between survivors and non-survivors

Variables at PICU admission	Survivors (***n*** = 56)	Nonsurvivors (***n*** = 11)	Total (***n*** = 67)	***p*** value
Age, mo, median (IQR)	18 (11, 38)	20(7.5, 41.5)	18 (10, 38.5)	0.889
Male gender, n (%)	32 (57.14%)	8 (72.7%)	40 (59.7%)	0.335
PRSM III score, median (IQR)	13 (10, 15)	14 (11, 18)	13 (10, 15)	0.133
days of illness before at PICU admission, median (IQR)	9 (7, 10.5)	6 (5, 12)	9 (6, 11)	0.959
Laboratory values, median (IQR)				
White blood cell, 109/L	6.55 (4.36,12.23)	6.11 (4.35, 10.87)	6.48 (4.29, 11.96)	0.923
Platelet, 109/L	254 (179, 349.5)	258 (148.5, 444.5)	258 (178, 353)	0.837
NK cells, %	3.85 (2.23, 6.57)	5 (2, 6.9)	4.06 (2.19, 6.68)	0.787
pH	7.4 (7.33, 7.44)	7.33 (7.28, 7.42)	7.4 (7.33, 7.44)	0.109
PaO2, mmHg	71 (57, 82.5)	73 (65.38, 84.38)	72 (57, 84)	0.853
PaCO2, mm Hg	43 (38, 55)	52 (44.5, 68)	44 (38, 55.5)	0.104
MAP, mmHg	55 (52, 68.25)	49 (42.5, 57)	55 (51.5, 67)	0.171
LA, mmol/L	1.95 (1.38, 2.33)	2 (1.65, 4.25)	2 (1.4, 2.6)	0.968
CI, L/min/m^2^	4.2 (3.9, 5)	3.6 (3.45, 4.1)	4.2 (3.85, 4.85)	0.011
TBIL,umol/L	4.42 (3.2, 5.87)	10.85 (5.35, 20.22)	5.02 (3.29, 6.61)	0.069
serum creatinine, umol/L	32.5 (25.5, 40.88)	34.5 (29.25, 54)	33 (26.25, 42)	0.291

*IQR* interquartile range, *NK cells* natural kill cell; CI: cardiac index, *MAP* mean arterial pressure, *TBIL* total bilirubin, *LA* Lactate

All patients were admitted to PICU for the reasons of fever (100%) and respiratory symptoms consistent with cough (100%) or whoop (65.7%), tachypnea (100%), acute respiratory failure (100%) requiring oxygenation support.

At PICU admission, co-infection (defined as pneumonia caused by adeno virus as well as typical bacteria, mycoplasma pneumoniae and other viruses) was seen in 25.37% (17/67) patients. During the PICU stay, nosocomial infection including VAP and bloodstream infection were seen in 32.84% (22/67) patients, higher morbidity in non-survival (63.6%,7/11) than in survival (26.78%,15/56). Nosocomial infected pathogens were isolated from different specimens including blood, sputum, bronchoalveolar lavage fluid, and hydrothorax. The most frequently isolated pathogens were *Acinetobacter baumanii* 9 patients (13.4%), *Klebsiella pneumoniae* in 7(10.5%), *mycoplasma pneumoniae* in 6 (9.8%), *Stenotrophomonas maltophilia* in 4 (5.9%), and *Candida albicans* in 3 (4.5%) (Table 2).

**Table 2:** PICU therapeutic interventions between survivors and non-survivors

	Survivors (***n*** = 56)	Non-survivors (***n*** = 11)	Total (***n*** = 67)	***p*** value
**Median of PICU stay, days**	11 (7.75, 18)	15 (11, 19.5)	11 (8, 18)	0.861
**Median of hospital stay, days**	22.5 (16, 34.25)	17 (16, 23.5)	22 (16, 31)	0.124
**Co-morbidity, n (%)**				
ARDS	27 (48.21%)	9 (81.82%)	36 (53.73%)	0.041
Liver dysfunction	21 (31.34%)	10 (90.9%)	31 (46.27%)	0.001
AKI	4 (7.14%)	5 (45.45%)	9 (13.43%)	0.001
shock	42 (75%)	10 (90.9%)	52 (77.61%)	0.247
GI dysfunction	31 (55.36%)	11 (100%)	42 (62.69%)	0.005
**PICU and hospital therapies, n (%)**				
Invasive Mechanical ventilation	51 (91.07%)	11 (100%)	62 (92.54%)	0.303
CRRT/RRT	11 (19.64%)	8 (72.73%)	19 (28.36%)	<0.001
ECMO	6 (10.71%)	3 (37.5%)	9 (13.43%)	0.141
Prone positioning, n (%)	15 (26.79%)	4 (36.36%)	19 (28.36%)	0.519
Neuromuscular blockade, n (%)	22 (39.29%)	8 (72.73%)	30 (44.78%)	0.041
Vasoactive use	48 (85.71%)	10 (90.9%)	58 (86.57%)	0.644
diuretics use	43 (76.79%)	8 (72.73%)	51 (76.12%)	0.773
Steroids use	55 (98.21%)	11 (100%)	66 (98.51%)	0.655
IV immunoglobulin	50 (89.29%)	10 (90.9%)	60 (89.55%)	0.872
Parenteral nutrition	12 (21.43%)	10 (90.9%)	22 (32.84%)	<0.001
Packed red blood cell perfusion	28 (50%)	10 (90.9%)	38 (56.72%)	0.012
**Nosocomial infenction**^**a**^**, n (%)**	15 (26.79%)	7 (63.64%)	22 (32.84%)	0.017
bacterial	15 (26.79%)	7 (63.64%)	22 (32.84%)	0.043
fungal	4 (7.14%)	1 (9.09%)	5 (7.46%)	0.687

^a^parts of patients complicated with bacteria, mycoplasma pneumoniae or fungi in nosocomial infection or co-infection

### Management and outcomes

All management decisions were performed by intensivist according to the guideline recommendation [8, 16, 17], experts’ opinion [18], and routine practice in our PICU. Additional oxygen was utilized in 100% (67cases) patients with 8.96% (6cases) requiring high flow nasal oxygen therapy, and 92.54% (62cases) requiring mechanical ventilation at some period during hospitalization. The indications for CRRT/RRT were:1) AKI which was defined according to the KDIGO criteria [19]; 2) Fluid overload which was defined as the fluid overload > 10% [fluid overload = (CRRT initial weight-PICU admission weight)/PICU admission weight× 100%] [20, 21]. The indications for ECMO were:1) severe hypoxemia with a PaO2/ FiO2 ratio of < 50 mmHg for > 3 h or < 80 mmHg for > 6 h, or pH < 7.25 and a partial pressure of arterial CO2 of ≥60 mmHg for > 6 h [22]. 2) hypoxemia complicated with cardio dysfunction when cardiac index (CI) less than 2.2 L/min.m2; and 3) hypoxemia complicated with circulatory dysfunction when persistent lactatemia greater than 4 mmol/L and vasoactive inotropic score (VIS) greater than 50.VIS was calculated as ([(epinephrine+ norepinephrine) ug/kg.min] × 100 + [(dobutamine + dopamine) ug/kg.min] + [milrinone ug/kg.min] × 15. Intravenous neuromuscular blockade was started if the peak inspiratory pressures approximated 28–30 cmH2O, and the patient was hypoxemic and continued to show excessive work of breathing despite adequate sedation [23]. Other cluster therapies included prone positioning, IV immunoglobulin, parenteral nutrition, vasoactive drugs, packed red blood cell perfusion, steroids (methylprednisolone 0.5–2.0 mg/kg.d for 3–5 days), diuretics, and antibiotics if needed (see in Table 2).

Among the 67 patients with severe adenovirus pneumonia, 11(16.42%) children died in PICU and among them, 10 (14.93%) cases died within 28 day after PICU admission. The overall PICU mortality was 16.42% (11/67), and 28-day mortality was14.93% (10/67). Patients aged less 2-year old accounted for 72.73% (8/11) of non-survivors.

The median lengths of stay in the PICU and hospital were 11 days (8, 18 days) and 22 days (16, 31 days), respectively (Table 2). The median duration of mechanical ventilation was 5.75 days (3.98, 11.67) in patients required invasive ventilation. In non-survivors, the median ventilator days was longer than that in survivors but without statistical significance (8.48 [4.77, 11.82] days vs. 10.75 [8.17, 19.08] days, *p* = 0.348). The rate of CRRT and use of neuromuscular blockade, parenteral nutrition, or packed red blood cell perfusion were significantly higher in non-survivors than that in survivors (*p* < 0.001, *p* = 0.041, *p* < 0.001, *p* = 0.012, respectively; Table 2). Moreover, the ratio of nosocomial infection was higher in non-survivors compared with survivors (63.64% vs. 26.79%, *p* = 0.017; Table 2).

The changes of parameters about ventilator characteristics and blood gas analysis in survivors and non-survivors were shown in Table 3. There were no significant differences in parameters including peak inspiratory pressure (PIP), positive expiratory end pressure (PEEP) and mean airway pressure (MAP) on the initial day, 3rd day and 7th day of invasive ventilation between survivors and non -survivors (all *p* > 0.05, Table 3). However, the values of Cydn on 3rd day and 7th day of invasive ventilation were significantly lower in non-survivors compared with survivors (*p* = 0.012, *p* = 0.045, Table 3). In addition, the ratio of PaO_2_/ FiO_2_ and SaO_2_ levels displayed a tendency decrease in non-survivors compared with survivors on the 3rd day after receiving invasive mechanical ventilation (*p* = 0.038, *p* = 0.008, respectively; Table 3).

**Table 3:** The changes of parameters about patients receiving invasive mechanical ventilation

Variables	D1			D3			D7		
	Survivor (*n* = 56)	Nonsurvivor (*n* = 11)	*p*-value	Survivors (*n* = 56)	Nonsurvivor (*n* = 11)	*P*-value	Survivor (*n* = 56)	Nonsurvivor (*n* = 11)	*p*-value
**PIP,cmH**_**2**_**O**	21 (19,23.5)	25 (21, 27)	0.145	21.5 (20, 24)	24 (20.5, 28)	0.377	22 (20, 26)	23 (21.75, 30)	0.27
**MAP,cmH**_**2**_**O**	11 (10, 12.5)	12 (10.5, 13.5)	0.372	11.5 (9.25, 13)	12 (11, 14.5)	0.412	12 (11.5, 14.5)	13 (11, 17.25)	0.458
**PEEP, cm H**_**2**_**O**	5 (4, 5)	5 (4, 5)	0.368	5 (5, 6)	5 (5, 5.5)	0.801	5 (5, 6)	6 (5, 6)	0.567
**Cdyn, cm H**_**2**_**O/kg**	0.4 (0.32, 0.48)	0.33 (0.31, 0.425)	0.286	0.52 (0.43, 0.55)	0.4 (0.31, 0.4)	0.012	0.5 (0.4, 0.56)	0.26 (0.22, 0.34)	0.045
**Tidal volume, ml/kg**	8 (7.8, 8)	7.2 (6.65, 8)	0.0029	8.3 (8, 8.5	8 (7.2, 8.1)	0.072	8.2 (8, 8.5)	7.55 (6.03, 8.35)	0.307
**PaO**_**2**_**/FiO**_**2**_**ratio, mmHg**	151 (113, 180.25)	140 (90, 143.5)	0.177	185 (139, 224)	111 (103,174.5)	0.038	170 (110, 212)	137.5 (89.5, 157.75)	0.384
Oxygenation index									
**PaCO**_**2**_**, mm Hg**	44 (35, 50.25)	48 (41.5, 57.5)	0.138	45 (40.5, 48.5)	42 (37.5, 55.5)	0.727	44 (41, 48)	58 (43, 69.5)	0.305
**PaO**_**2**_**, mm Hg**	72 (57, 86.25)	73 (58.375, 84.5)	0.871	78 (72.5, 89.5)	78 (72.5, 89.5)	0.087	77 (55, 90)	65 (51.5, 75)	0.357
**SaO**_**2**_**, %**	95 (92, 96)	93 (90.5, 95)	0.265	96 (95, 97)	93 (91.5, 95.5)	0.008	96.5 (92.25, 98)	91 (89, 95.5)	0.655

D1: initial day of invasive mechanical ventilation; D3: 3 days of ventilation; D7: 7 days of ventilation; PIP: Cdyn: lung dynamic compliance; *MAP* mean airway pressure

Besides the lower cardiac index (CI) in non-survivors than survivors at PICU admission (Table 1), CD4^+^ cells percentage showed a higher tendency in non-survivors than that in survivors at PICU admission (*p* = 0.071, Table 4). There were no significant differences in aspects of white blood cell and platelet count, NK cell, CD4^+^, CD8^+^, CD19^+^ percentage between two groups at PICU admission. During PICU stay, platelet Count was significantly lower in non-survivors at 7 days after PICU admission when compared with survivors (93 [85, 371], vs. 327 [257.75, 443.75] × 10^9^/L, *p* = 0.039; Table 4). In addition, the interleukin 6 (IL-6) and IL-10 were significantly higher in non-survivors than those of survivors at 7 days after PICU admission (*p* = 0.035, *p* < 0.01, respectively; Table 4).

**Table 4:** Changes of blood cell and immunological parameters at PICU admission and 7 days after admission

Variables	PICU admission			7 days after admission		
	Survivors (***n*** = 56)	Non-survivors (***n*** = 11)	***P*** value	Survivors (***n*** = 56)	Non-survivors (***n*** = 11)	***P*** value
Hb, g/L	101.5 (90, 113.5)	100 (100, 109.5)	0.699	99.5 (95, 106)	95 (90, 110)	0.457
WBC, 10^9^/L	6.55 (4.36, 12.23)	6.11 (4.35, 10.87)	0.923	8.78 (5.26, 10.77)	4.37 (3.39, 11.69)	0.774
platlat,10^9^/L	254 (179, 349.5)	258 (148.5, 444.5)	0.837	327 (257.75, 443.75)	93 (85, 371)	0.039
NK cells, %	3.85 (2.23, 6.57)	5 (2, 6.895)	0.787	3.98 (2.185, 6.01)	2.98 (1.02, 8.08)	0.812
CD19+, %	44.02 (30.91, 52.64)	42.1 (24.47, 53.55)	0.437	34.58 (30.13, 45.36)	38.58 (24.66, 44.04)	0.804
CD4+, %	25.56 (19.5, 31.39)	32.48 (23.2, 41.59)	0.071	27.49 (22.96, 35.48)	33.23 (29.82, 38.79)	0.489
CD8+, %	20.02 (14.96, 26.75)	16.36 (15.34 20.25)	0.555	20.14 (17.76, 28.28)	20.58 (14.23, 22.5)	0.614
IL-6, ng/L	0.1 (0.1, 0.1)	0.1 (0.1,0.1)	0.449	0.1 (0.1, 0.1)	47.77 (0.1, 239.29)	0.035
IL-8, ng/L	0.1 (0.1, 12.09)	0.1 (0.1, 36.39)	0.707	0.1 (0.1, 0.1)	7.15 (3.09, 20.21)	0.144
IL-10, ng/L	0.1 (0.1, 4.05)	8.99 (0.1, 32.57)	0.855	0.1 (0.1, 0.1)	18.39 (0.1, 32.74)	0.0005
IL-2R, ug/L	15.31 (4.27, 28.78)	18.92 (11.38, 33.37)	0.459	10.25 (20.81, 24.32)	21.71 (16.37 33.43)	0.089

### Multivariate logistic analysis

By univariate logistic analysis, the patients were associated with worse outcome of severe adenovirus pneumonia in complicated liver dysfunction due to the adeno virus infection (16.485 [1.745 ~ 155.705], *p* = 0.014), AKI (10.833[2.269 ~ 51.706], *p* = 0.003), gastrointestinal dysfunction (0.355 [0.178 ~ 0.706], *p* = 0.003) which was defined according to the European Consensus Definition of acute gastrointestinal injury (AGI) [24], encephalopathy (5.629 [1.333 ~ 23.774], *p* = 0.019), co-infection & nosocomial infection (15.455 [1.847 ~ 129.326], *p* = 0.012) (Table 5). By multivariate logistic regression analysis, the independently risk factor associated with mortality was liver dysfunction (21.231 [1.696 ~ 265.779], *p* = 0.018) and nosocomial infection (2.574 [0.986 ~ 15.671], *p* = 0.05) (Table 5).

**Table 5:** Logistic analysis of variables independently associated with hospital mortality

Outcome	OR	St.Err.	95%CI	*P* value
**Univariate logistic regression**				
PRISM III	1.079	0.119	0.868 ~ 1.342	0.690
ARDS	0.5	0.185	0.242 ~ 1.032	0.061
Liver dysfunction	16.485	18.887	1.745 ~ 155.705	0.014
AKI	10.833	8.639	2.269 ~ 51.706	0.003
Shock	3.333	3.644	0.391 ~ 28.41	0.271
Gastrointestinal dysfunction	0.355	0.124	0.178 ~ 0.706	0.003
Encephalopathy	5.629	4.138	1.333 ~ 23.774	0.019
Co-infection& nosocomial infection	15.455	16.751	1.847 ~ 129.326	0.012
**Multivariate logistic regression**				
Liver dysfunction	21.231	27.376	1.696 ~ 265.779	0.018
nosocomial infection	2.574	0.706	0.986 ~ 15.671	0.05

## Discussion

This is the first REPORT describing overall morbidity and mortality for pediatric patients with severe adenoviral pneumonia admitted to the PICU in mainland China. In our PICU 3-year period, the hospital all-cause hospital mortality of severe community acquired adenoviral pneumonia was 16.42%, and 28-day mortality (deaths after discharge from hospital were not included) was 14.93%. In the 11 non-survivor patients, 3 of them died from liver dysfunction due to adeno virus infection, 3 died from refractory hypoxic respiratory failure, two of them died from refractory septic shock caused by nosocomial infection of *Klebsiella pneumoniae* and Stenotrophomonas maltophili, and one patient died from intracranial bleeding. We identified the independently risk factors for mortality including patients complicated with liver dysfunction and nosocomial infection.

Adenovirus disease is a self-limiting in majority of immunocompetent population, but can cause life-threatening illness in immunocompromised hosts [25–27]. Adenovirus accounts for at least 5 to 10% of pediatric respiratory tract infections in children [1, 2]. The overall PICU hospitalization with severe adenoviral pneumonia in the present study was 9.99% of CAP. More importantly, the cases number from 2016 to 2019 was with increased tendency in our PICU, especially with a higher incidence rate between 2018 to 2019. Most patients with adenovirus infection are younger than 2-year old [56/67,83.6%]. When severe adenovirus pneumonia progressed with MODS, the mortality is higher over 50% [5]. In the present study, children aged < 24 months accounted for 72.73% of total deaths. There are limited antiviral drugs available for adenovirus. Cidofovir is an antiviral drug which use has been associated with significant reductions of adenovirus load and, in some series improved survival in reports [10, 28]. Until recently, Cidofovir is not available in China till 2019, and has been neither widely used in children, nor has it been used in our cases. All these results suggested that adenovirus pneumonia requires our attention due to the high mortality involved, especially in China where there have no specific anti-adenovirus drugs or vaccine for children until now.

Mechanical ventilation remains the main stay of management. For the hypoxemia respiratory failure/ARDS ventilated patients caused by adenovirus in this study, the PaO_2_/FiO_2_ ratio at initial presentation was relatively low in survivors (151[interquartile range:113, 180.25]) and non-survivors (140[interquartile range:107, 143.5]).The PaO_2_/FiO_2_ was no statistical difference at initial day, 3rd day and 7th day ventilation between survivors and non-survivors. But lower Cdyn at 3rd day and 7th day ventilation in non-survivors (*p* = 0.012, *p* = 0.045). In order to ensure the mechanical ventilation and to improve the level of PaO_2_/FiO_2_, we used prone position and neuromuscular blockers in appropriate patients. There was no difference in the proportion of prone position between the two groups (*p* = 0.519), but the proportion of neuromuscular blockers was significantly higher in non-survivors than that in survivors (*p* = 0.041).

Under 2-year old could partially contribute to the high incidence of severe adenovirus pneumonia and high mortality [2, 24–26]. Adenovirus-induced immunosuppression might augment the susceptibility to nosocomial microbial infections. In this retrospective study, the high levels of IL-6 and IL-10 in non-survivors were measured, and we identified that the nosocomial infection after PICU admission was an independent risk factors for all-cause hospital mortality. This indicated that high levels of IL-6 and IL-10 in non-survivors could provide an insight for adenovirus-associated nosocomial infection. IL-6 plays a role in immunosuppression by driving differentiation of myeloid suppressor cells together with TGF-β in cancer pathogenesis [29]. Otherwise, IL-10 is produced by Treg cells and Th2-type cells and suppresses the Th1 response [30]. The continued release of IL-10 contributes to sepsis-induced immunosuppression resulting in more susceptibility to nosocomial infection [31, 32]. Whether high levels of IL-6 and IL-10 in patients with adenovirus infection contribute the worse outcome warrants further investigation.

ECMO support for severe adenoviral infection has been reported in several studies [10, 33, 34]. Retrospective data from the extracorporeal life support organization (ELSO) registry showed that pediatric patients with AV infection supported with ECMO, had a survival to hospital discharge of 38% which was even lower in neonates [8]. B More recently, Ramanathan et al. observed over the last 25 years ELSO registry across all age groups who needed ECMO for severe adenoviral pneumonia in neonatal, pediatric, and adult patients, the hospital mortality was 58% with no significant improvement from 1992 to 2016 [12]. In our study, 6 patients survived in whom (9cases) received ECMO support from 2016 to 2019. Our results suggest that ECMO as the last rescue treatment for severe adenoviral pneumonia, is worthy of further exploration.

Our study has several limitations. First, it is a retrospective analysis from single PICU, we didn’t include those adeno virus pneumonia not requiring PICU admission, which contributed to the power of our study is limited by the small size of case series. Second, we didn’t detect of adenovirus serotype, which might affect the judgment of the outcomes. Third, long-term follow-up data was unavailable.

## Conclusion

Our study demonstrated that adenovirus pneumonia remains a major cause of morbidity and mortality in the PICU. We identified several factors with higher mortality, including complicated with shock, liver dysfunction, AKI, gastrointestine dysfunction, encephalopathy, and co-infection& nosocomial infection. The patients complicated with liver dysfunction and associated nosocomial infection were independent risk factors for mortality.

## Data Availability

Our present study was a retrospective observational study. All the data were obtained from medical records of patients. The datasets used and/or analysed during the current study are not publically available, but they can be shared from the corresponding author on reasonable request except for the identifying/confidential patient data.

## Abbreviations

PICU: Pediatric intensive care unit
CAP: Community acquired pneumonia
ARDS: Acute respiratory distress syndrome
MODS: Multiple organ dysfunction syndrome
RT-PCR: Real-time polymerase chain reaction
ECMO: Extracorporeal membrane oxygenation
CRRT/RRT: Continuous renal replacement therapy/ renal replacement therapy
PaO_2_/FiO_2_: The ratio of the partial pressure of oxygen in arterial blood (PaO2) to the inspired oxygen fraction (FiO2)
PRISM III: Pediatric risk of mortality III
Cdyn: Lung dynamic compliance
CI: Cardiac index
MAP: Mean arterial pressure
TBIL: Total bilirubin
LA: Lactic acid
sCr: serum creatinine
PLT: Platelet counts
NK: Natural kill cell
IQR: Interquartile range
ORs: Odd ratios
AKI: Acute kidney injury
ELSO: Extracorporeal life support organization
